# Preparedness for a first clinical placement in nursing: a descriptive qualitative study

**DOI:** 10.1186/s12912-024-01916-x

**Published:** 2024-05-22

**Authors:** Philippa H. M. Marriott, Jennifer M. Weller-Newton, Katharine J. Reid

**Affiliations:** 1 Department of Nursing, The University of Melbourne, Grattan St, Parkville, VIC 3010 Australia; 2 Department of Rural Health, The University of Melbourne, Grattan St, Shepparton, VIC 3630 Australia; 3 Present Address: School of Nursing and Midwifery, University of Canberra, Kirinari Drive, Bruce, Canberra, ACT 2617 Australia; 4 Present address: Department of Medical Education, Melbourne Medical School, The University of Melbourne, Grattan St, Parkville, VIC 3010 Australia

**Keywords:** Postgraduate nursing education, Nursing students, Nursing schools, Clinical skills, Qualitative research

## Abstract

**Background:**

A first clinical placement for nursing students is a challenging period involving translation of theoretical knowledge and development of an identity within the healthcare setting; it is often a time of emotional vulnerability. It can be a pivotal moment for ambivalent nursing students to decide whether to continue their professional training. To date, student expectations prior to their first clinical placement have been explored in advance of the experience or gathered following the placement experience. However, there is a significant gap in understanding how nursing students’ perspectives about their first clinical placement might change or remain consistent following their placement experiences. Thus, the study aimed to explore first-year nursing students’ emotional responses towards and perceptions of their preparedness for their first clinical placement and to examine whether initial perceptions remain consistent or change during the placement experience.

**Methods:**

The research utilised a pre-post qualitative descriptive design. Six focus groups were undertaken before the first clinical placement (with up to four participants in each group) and follow-up individual interviews (*n* = 10) were undertaken towards the end of the first clinical placement with first-year entry-to-practice postgraduate nursing students. Data were analysed thematically.

**Results:**

Three main themes emerged: (1) adjusting and managing a raft of feelings, encapsulating participants’ feelings about learning in a new environment and progressing from academia to clinical practice; (2) sinking or swimming, comprising students’ expectations before their first clinical placement and how these perceptions are altered through their clinical placement experience; and (3) navigating placement, describing relationships between healthcare staff, patients, and peers.

**Conclusions:**

This unique study of first-year postgraduate entry-to-practice nursing students’ perspectives of their first clinical placement adds to the extant knowledge. By examining student experience prior to and during their first clinical placement experience, it is possible to explore the consistency and change in students’ narratives over the course of an impactful experience. Researching the narratives of nursing students embarking on their first clinical placement provides tertiary education institutions with insights into preparing students for this critical experience.

## Background

First clinical placements enable nursing students to develop their professional identity through initial socialisation, and where successful, first clinical placement experiences can motivate nursing students to persist with their studies [1–4]. Where the transition from the tertiary environment to learning in the healthcare workplace is turbulent, it may impact nursing students’ learning, their confidence and potentially increase attrition rates from educational programs [2, 5, 6]. Attrition from preregistration nursing courses is a global concern, with the COVID-19 pandemic further straining the nursing workforce; thus, the supply of nursing professionals is unlikely to meet demand [7]. The COVID-19 pandemic has also impacted nursing education, with student nurses augmenting the diminishing nursing workforce [7, 8].

The first clinical placement often triggers immense anxiety and fear for nursing students [9, 10]. Research suggests that among nursing students, anxiety arises from perceived knowledge deficiencies, role ambiguity, the working environment, caring for ‘real’ people, potentially causing harm, exposure to nudity and death, and ‘not fitting in’ [2, 3, 11]. These stressors are reported internationally and often relate to inadequate preparation for entering the clinical environment [2, 10, 12]. Previous research suggests that high anxiety before the first clinical placement can be related to factors likely to affect patient outcomes, such as self-confidence and efficacy [13]. High anxiety during clinical placement may impair students’ capacity to learn, thus compromising the value of the clinical environment for learning [10].

The first clinical placement often occurs soon after commencing nursing training and can challenge students’ beliefs, philosophies, and preconceived ideas about nursing. An experience of cultural or ‘reality’ shock often arises when entering the healthcare setting, creating dissonance between reality and expectations [6, 14]. These experiences may be exacerbated by tertiary education providers teaching of ‘ideal’ clinical practice [2, 6]. The perceived distance between theoretical knowledge and what is expected in a healthcare placement, as opposed to what occurs on clinical placement, has been well documented as the theory-practice gap or an experience of cognitive dissonance [2, 3].

Given the pivotal role of the first clinical placement in nursing students’ trajectories to nursing practice, it is important to understand students’ experiences and to explore how the placement experience shapes initial perceptions. Existing research focusses almost entirely either on describing nursing students’ projected emotions and perceptions prior to undertaking a first clinical placement [3] or examines student perceptions of reflecting on a completed first placement [15]. We wished to examine consistency and change in student perception of their first clinical placement by tracking their experiences longitudinally. We focused on a first clinical placement undertaken in a Master of Nursing Science. This two-year postgraduate qualification provides entry-to-practice nursing training for students who have completed any undergraduate qualification. The first clinical placement component of the course aimed to orient students to the clinical environment, support students to acquire skills and develop their clinical reasoning through experiential learning with experienced nursing mentors.

This paper makes a significant contribution to understanding how nursing students’ perceptions might develop over time because of their clinical placement experiences. Our research addresses a further gap in the existing literature, by focusing on students completing an accelerated postgraduate two-year entry-to-practice degree open to students with any prior undergraduate degree. Thus, the current research aimed to understand nursing students’ emotional responses and expectations and their perceptions of preparedness before attending their first clinical placement and to contrast these initial perceptions with their end-of-placement perspectives.

## Methods

### Study design

A descriptive qualitative study was undertaken, utilising a pre- and post-design for data collection. Focus groups with first-year postgraduate entry-to-practice nursing students were conducted before the first clinical placement, with individual semi-structured interviews undertaken during the first clinical placement.

### Setting and participants

All first-year students enrolled in the two-year Master of Nursing Science program (*n* = 190) at a tertiary institution in Melbourne, Australia, were eligible to participate. There were no exclusion criteria. At the time of this study, students were enrolled in a semester-long subject focused on nursing assessment and care. They studied the theoretical underpinnings of nursing and science, theoretical and practical nursing clinical skills and Indigenous health over the first six weeks of the course. Students completed a preclinical assessment as a hurdle before commencing a three-week clinical placement in a hospital setting, a subacute or acute environment. Overall, the clinical placement aimed to provide opportunities for experiential learning, skill acquisition, development of clinical reasoning skills and professional socialisation [16, 17].

In total, sixteen students participated voluntarily in a focus group of between 60 and 90 min duration; ten of these students also participated in individual interviews of between 30 and 60 min duration, a number sufficient to reach data saturation. Table 1 shows the questions used in the focus groups conducted before clinical placement commenced and the questions for the semi-structured interview questions conducted during clinical placement. Study participants’ undergraduate qualifications included bachelor’s degrees in science, arts and business. A small number of participants had previous healthcare experience (e.g. as healthcare assistants). The participants attended clinical placement in the Melbourne metropolitan, Victorian regional and rural hospital locations.

**Table 1:** Focus group and interview questions utilised in the study

Before clinical placement: Focus group questions	During clinical placement: Semi-structured interview questions
Describe how you are feeling about starting your clinical placement? What do you expect for your first clinical placement? What do you think it will be like? What aspects of your preparation for this clinical placement were helpful? Were there any aspects of your preparation for this clinical placement that were unhelpful? What knowledge, skills and attitudes do you believe are important for you to have prior to starting your first placement? How do you see your role as student nurse?	Now you have undertaken your clinical placement, on reflection how prepared were you? Do you feel you have developed an understanding of what is expected of you as a student nurse? Was this clinical placement what you expected it to be? How do you see your role as a student nurse? Has the placement changed the way you feel? What aspects of your preparation for this clinical placement were helpful? Were there any aspects of your preparation for this clinical placement that were unhelpful? What knowledge, skills and attitudes do you believe are important for you to have prior to starting your first placement? If you had the chance to give advice to the Department of Nursing on preparing students for clinical placement, what advice would you give? Were there any experiences during the first placement that made you more confident in terms of progressing with nursing as a profession?

### Data collection

The study comprised two phases. The first phase comprised six focus groups prior to the first clinical placement, and the second phase comprised ten individual semi-structured interviews towards the end of the first clinical placement. Focus groups (with a maximum of four participants) and individual interviews were conducted by the lead author online via Zoom and were audio-recorded. Capping group size to a relatively small number considered diversity of perceptions and opportunities for participants to share their insights and to confirm or contradict their peers, particularly in the online environment [18, 19].

Focus groups and interview questions were developed with reference to relevant literature, piloted with volunteer final-year nursing students, and then verified with the coauthors. All focus groups and interviewees received the same structured questions (Table 1) to ensure consistency and to facilitate comparison across the placement experience in the development of themes. Selective probing of interviewees’ responses for clarification to gain in-depth responses was undertaken. Nonverbal cues, impressions, or observations were noted.

### Rigour

The lead author was a registered nurse who had a clinical teaching role within the nursing department and was responsible for coordinating clinical placement experiences. To ensure rigour during the data collection process, the lead author maintained a reflective account, exploring her experiences of the discussions, reflecting on her interactions with participants as a researcher and as a clinical educator, and identifying areas for improvement (for instance allowing participants to tell their stories with fewer prompts). These reflections in conjunction with regular discussion with the other authors throughout the data collection period, aided in identifying any researcher biases, feelings and thoughts that possibly influenced the research [20].

To maintain rigour during the data analysis phase, we adhered to a systematic process involving input from all authors to code the data and to identify, refine and describe the themes and subthemes reported in this work. This comprehensive analytic process, reported in detail in the following section, was designed to ensure that the findings arising from this research were derived from a rigorous approach to analysing the data.

### Data analysis

Focus groups and interviews were transcribed using the online transcription service Otter (https://otter.ai/) and then checked and anonymised by the first author. Preliminary data analysis was carried out simultaneously by the first author using thematic content analysis proposed by Braun and Clarke [21] using NVivo 12 software [22]. All three authors undertook a detailed reading of the first three transcripts from both the focus groups and interviews and independently identified major themes. This preliminary coding was used as the basis of a discussion session to identify common themes between authors, to clarify sources of disagreement and to establish guidelines for further coding. Subsequent coding of the complete data set by the lead author identified a total of 533 descriptive codes; no descriptive code was duplicated across the themes. Initially, the descriptive codes were grouped into major themes identified from the literature, but with further analysis, themes emerged that were unique to the current study.

The research team met frequently during data analysis to discuss the initial descriptive codes, to confirm the major themes and subthemes, to revise themes on which there was disagreement and to identify any additional themes. Samples of quotes were reviewed by the second and third authors to decide whether these quotes were representative of the identified themes. The process occurred iteratively to refine the thematic categories, to discuss the definitions of each theme and to identify exemplar quotes.

### Ethical considerations

The lead author was a clinical teacher and the clinical placement coordinator in the nursing department at the time of the study. Potential risks of perceived coercion and power imbalances were identified because of the lead author’s dual roles as an academic and as a researcher. To manage these potential risks, an academic staff member who was not part of the research study informed students about the study during a face-to-face lecture and ensured that all participants received a plain language statement identifying the lead author’s role and how perceived conflicts of interest would be managed. These included the lead author not undertaking any teaching or assessment role for the duration of the study and ensuring that placement allocations were completed prior to undertaking recruitment for the study. All students who participated in the study provided informed written consent. No financial or other incentives were offered. Approval to conduct the study was granted by the University of Melbourne Human Research Ethics Committee (Ethics ID 1955997.1).

## Results

Three main themes emerged describing students’ feelings and perceptions of their first clinical placement. In presenting the findings, before or during has been assigned to participants’ quotes to clarify the timing of students’ perspectives related to the clinical placement.

### Major theme 1: Adjusting and managing a raft of feelings

The first theme encompassed the many positive and negative feelings about work-integrated learning expressed by participants before and during their clinical placement. Positive feelings before clinical placement were expressed by participants who were comfortable with the unknown and cautiously optimistic.

*I am ready to just go with the flow, roll with the punches* (Participant [P]1 before).

Overwhelmingly, however, the majority of feelings and thoughts anticipating the first clinical placement were negatively oriented. Students who expressed feelings of fear, anxiety, lack of knowledge, lack of preparedness, uncertainty about nursing as a career, or strong concerns about being a burden were all classified as conveying negative feelings. These negative feelings were categorised into four subthemes.

### Subtheme 1.1 I don’t have enough knowledge

All participants expressed some concerns and anxiety before their first clinical placement. These encompassed concerns about knowledge inadequacy and were linked to a perception of under preparedness. Participants’ fears related to harming patients, responsibility for managing ‘real’ people, medication administration, and incomplete understanding of the language and communication skills within a healthcare setting. Anxiety for many participants merged with the logistics and management of their life during the clinical placement.

*I’m scared that they will assume that I have more knowledge than I do* (P3 before).

*I feel quite similar with P10, especially when she said fear of unknown and fear that she might do something wrong* (P9 before).

### Subtheme 1.2 Worry about judgment, being seen through that lens

Participants voiced concerns that they would be judged negatively by patients or healthcare staff because they perceived that the student nurse belonged to specific social groups related to their cultural background, ethnicity or gender. Affiliation with these groups contributed to students’ sense of self or identity, with students often describing such groups as a community. Before the clinical placement, participants worried that such judgements would impact the support they received on placement and their ability to deliver patient care.

*Some older patients might prefer to have nurses from their own background, their own ethnicity, how they would react to me, or if racism is involved* (P10 before).

*I just don’t want to reinforce like, whatever negative perceptions people might have of that community* (P16 before).

Participants’ concerns prior to the first clinical placement about judgement or poor treatment because of patients’ preconceived ideas about specific ethnic groups did not eventuate.

*I mean, it didn’t really feel like very much of a thing once I was actually there. It is one of those things you stress about, and it does not really amount to anything* (P16 during).

Some students’ placement experiences revealed the positive benefits of their cultural background to enhancing patient care. One student affirmed that the placement experience reinforced their commitment to nursing and that this was related to their ability to communicate with patients whose first language was not English.

*Yeah, definitely. Like, I can speak a few dialects. You know, I can actually see a difference with a lot of the non-English speaking background people. As soon as you, as soon as they’re aware that you’re trying and you’re trying to speak your language, they, they just open up. Yeah, yes. And it improves the care* (P10 during).

However, a perceived lack of judgement was sometimes attributed to wearing the full personal protective equipment required during the COVID-19 pandemic, which meant that their personal features were largely obscured. For this reason, it was more difficult for patients to make assumptions or attributions about students’ ethnic or gender identity based on their appearance.

*People tend to assume and call us all girls, which was irritating. It was mostly just because all of us were so covered up, no one could see anyone’s faces* (P16 during).

### Subtheme 1.3 Is nursing really for me?

Prior to their first clinical placement experience, many participants expressed ambivalence about a nursing career and anticipated that undertaking clinical placement could determine their suitability for the profession. Once exposed to clinical placement, the majority of students were completely committed to their chosen profession, with a minority remaining ambivalent or, in rare cases, choosing to leave the course. Not yet achieving full commitment to a nursing career was related to not wishing to work in the ward they had for their clinical placement, while remaining open to trying different specialities.

*I didn’t have an actual idea of what I wanted to do after arts, this wasn’t something that I was aiming towards specifically* (P14 before).

*I think I’m still not 100%, but enough to go on, that I’m happy to continue the course as best as I can* (P11 during).

### Subtheme 1.4 Being a burden

Before clinical placement, participants had concerns about being burdensome and how this would affect their clinical placement experiences.

*If we end up being a burden to them, an extra responsibility for them on top of their day, then we might not be treated as well* (P10 before).

A sense of burden remained a theme during the clinical placement for participants for the first five to seven days, after which most participants acknowledged that their role became more active. As students contributed more productively to their placement, their feelings of being a burden reduced.

### Major theme 2: Sinking or swimming

The second major theme, sinking or swimming, described participants’ expectations about a successful placement experience and identified themes related to students’ successes (‘swimming’) or difficulties (‘sinking’) during their placement experience. Prior to clinical placement, without a realistic preview of what the experience might entail, participants were uncertain of their role, hoped for ‘nice’ supervising nurses and anticipated an observational role that would keep them afloat.

*I will focus on what I want to learn and see if that coincides with what is expected, I guess* (P15 before).

During the clinical placement, the reality was very different, with a sense of sinking. Participants discovered, some with shock, that they were expected to participate actively in the healthcare team.

*I got the sense that they were not going to muck around, and, you know, they’re ‘gonna’ use the free labour that came with me* (P1 during).

Adding to the confusion about the expected placement experience, participants believed that healthcare staff were unclear about students’ scope of practice for a postgraduate entry-to-practice degree, creating misalignment between students’ and supervising nurses’ expectations.

*It seems to me like the educators don’t really seem to have a clear picture of what the scope is, and what is actually required or expected of us* (P10 during).

In exploring perceived expectations of the clinical placement and the modifying effect of placement on initial expectations, three subthemes were identified: translation to practice is overwhelming, trying to find the rhythm or jigsaw pieces, and individual agency.

### Subtheme 2.1 Translation to practice is overwhelming

Before clinical placement, participants described concerns about insufficient knowledge to enable them to engage effectively with the placement experience.

*If I am doing an assessment understanding what are those indications and why I would be doing it or not doing it at a certain time* (P1 before).

Integrating and applying theoretical content while navigating an unfamiliar clinical environment created a significant gap between theory and practice during clinical placement. As the clinical placement experience proceeded and initial fears dissipated, students became more aware of applying their theoretical knowledge in the clinical context.

*We’re learning all this theory and clinical stuff, but then we don’t really have a realistic idea of what it’s like until we’re kind of thrown into it for three weeks* (P10 during).

### Subtheme 2.2 Trying to find the rhythm or the jigsaw pieces

Before clinical placement, participants described learning theory and clinical skills with contextual unfamiliarity. They had the jigsaw pieces but did not know how to assemble it; they had the music but did not know the final song. When discussing their expectations about clinical placement, the small number of participants with a healthcare background (e.g. as healthcare assistants) proposed realistic answers, whereas others struggled to answer or cited stories from friends or television. With a lack of context, feelings of unpreparedness were exacerbated. Once in the clinical environment, participants further emphasised that they could not identify what they needed to know to successfully prepare for clinical placement.

*It was never really pieced together. We’ve learned bits and pieces, and then we’re putting it together ourselves* (P8 during).

*On this course I feel it was this is how you do it, but I did not know how it was supposed to be played, I did not know the rhythm* (P4 during).

### Subtheme 2.3 Individual agency

Participants’ individual agency, their attitude, self-efficacy, and self-motivation affected their clinical placement experiences. Participant perceptions in advance of the clinical placement experience remained consistent with their perspectives following clinical placement. Before clinical placement, participants who were highly motivated to learn exhibited a growth mindset [23] and planned to be proactive in delivering patient care. During their clinical placement, initially positive students remained positive and optimistic about their future. Participants who believed that their first clinical placement role would be largely observational and were less proactive about applying their knowledge and skills identified boredom and a lack of learning opportunities on clinical placement.

*A shadowing position, we don’t have enough skills and authority to do any work, not do any worthwhile skills* (P3 before).

*I thought it would be a lot busier, because we’re limited with our scope, so there’s not much we can do, it’s just a bit slower than I thought* (P12 during).

Individual agency appears to influence a successful first clinical placement; other factors may also be implicated but were not the focus of this study. Further research exploring the relationships between students’ age, life experience, resilience, individual agency, and the use of coping strategies during a first clinical placement would be useful.

### Major theme 3: The reality of navigating placement relationships

The third main theme emphasised the reality of navigating clinical placement relationships and explored students’ relationships with healthcare staff, patients, and peers. Before clinical placement, many participants, especially those with healthcare backgrounds, expressed fears about relationships with supervising nurses. They perceived that the dynamics of the team and the healthcare workplace might influence the support they received. Several participants were nervous about attending placement on their own without peers for support, especially if the experience was challenging. Participants identified expectations of being mistreated, believing that it was unavoidable, and prepared themselves to not take it personally.

*For me it’s where we’re going to land, are we going to be in a supportive, kind of nurturing environment, or is it just kind of sink or swim?* (P5 before).

*If you don’t really trust them, you’re nervous the entire time and you’ll be like what if I get it wrong* (P16 before).

Despite these concerns, students strongly emphasised the value of relationships during their first clinical placement, with these perceptions unchanged by their clinical placement experience. Where relationships were positive, participants felt empowered to be autonomous, and their self-confidence increased.

*You get that that instant reaction from the patients. And that makes you feel more confident. So that really got me through the first week* (P14 during).

*I felt like I was intruding, then as I started to build a bit of rapport with the people, and they saw that I was around, I don’t feel that as much now* (P1 during).

Such development hinged on the receptiveness and support of supervising nurses, the team on the ward, and patients and could be hindered by poor relationships.

*He was the old-style buddy nurse in his fifties, every time I questioned him, he would go ssshh, just listen, no questions, it was very stressful* (P10 during).

*It depends whether the buddy sees us as an extra pair of hands, or we’re learners* (P11 during).

Where students experienced poor behaviour from supervising nurses, they described a range of emotional responses to these interactions and also coping strategies including avoiding unfriendly staff and actively seeking out those who were more inclusive.

*If they weren’t very nice, it wouldn’t be very enjoyable and if they didn’t trust you, then it would be a bit frustrating, that like I can do this, but you won’t let me* (P12 during).

*If another nurse was not nice to me, and I was their buddy, I would literally just not buddy with them and go and follow whoever was nice to me* (P4 during).

Relationships with peers were equally important; students on clinical placement with peers valued the shared experience. In contrast, students who attended clinical placement alone at a regional or rural hospital felt disconnected from the opportunities that learning with peers afforded.

## Discussion

Our research explored the emotional responses and perceptions of preparedness of postgraduate entry-to-practice nursing students prior to and during their first clinical placement. In this study, we described how the perceptions of nursing students remained consistent or were modified by their clinical placement experiences. Our analysis of students’ experiences identified three major themes: adjusting and managing a raft of feelings; sinking or swimming; and the reality of navigating placement relationships. We captured similar themes identified in the literature; however, our study also identified novel aspects of nursing students’ experiences of their first clinical placement.

The key theme, adjusting and managing a raft of feelings, which encapsulates anxiety before clinical placement, is consistent with previous research. This theme included concerns in communicating with healthcare staff and managing registered nurses’ negative attitudes and expectations, in addition to an academic workload [11, 24]. Concerns not previously identified in the literature included a fear of judgement or discrimination by healthcare staff or patients that might impact the reputation of marginalised communities. Fortunately, these initial fears largely dissipated during clinical placement. Some students discovered that a diverse cultural background was an asset during their clinical placement. Although these initial fears were ameliorated by clinical placement experiences, evidence of such fears before clinical placement is concerning. Further research to identify appropriate support for nursing students from culturally diverse or marginalised communities is warranted. For example, a Finnish study highlighted the importance of mentoring culturally diverse students, creating a pedagogical atmosphere during clinical placement and integrating cultural diversity into nursing education [25].

Preclinical expectations of being mistreated can be viewed as an unavoidable phenomenon for nursing students [26]. The existing literature highlights power imbalances and hierarchical differences within the healthcare system, where student nurses may be marginalised, disrespected, and ignored [9, 27, 28]. During their clinical placement, students in our study reported unintentional incivility by supervising nurses: feeling not wanted, ignored, or asked to remain quiet by supervising nurses who were unfriendly or highly critical. These findings were similar to those of Thomas et al.’s [29] UK study and were particularly heightened at the beginning of clinical placement. Several students acknowledged that nursing staff fatigue from a high turnover of students on their ward and the COVID-19 pandemic could be contributing factors. In response to such incivility, students reported decreased self-confidence and described becoming quiet and withdrawing from active participation with their patients. Students oriented their behaviour towards repetitive low-level tasks, aiming to please and help their supervising nurse, to the detriment of learning opportunities. Fortunately, these incidents did not appear to impact nursing students’ overall experience of clinical placement. Indeed, students found positive experiences with different supervising nurses and their own self-reflection assisted with coping. Other active strategies to combat incivility identified in the current study that were also identified by Thomas et al. [29] included avoiding nurses who were uncivil, asking to work with nurses who were ‘nice’ to them, and seeking out support from other staff as a coping strategy. The nursing students in our study were undertaking a postgraduate entry-to-practice qualification and already had an undergraduate degree. The likely greater levels of experience and maturity of this cohort may influence their resilience when working with unsupportive supervising nurses and identifying strategies to manage challenging situations.

The theory-practice gap emerged in the theme of sinking or swimming. A theory-practice gap describes the perceived dissonance between theoretical knowledge and expectations for the first clinical placement, as opposed to the reality of the experience, and has been reported in previous studies (see, for instance, 24, 30–32). Existing research has shown that when the first clinical placement does not meet inexperienced student nurses’ expectations, a disconnect between theory and practice occurs, creating feelings of being lost and insecure within the new environment, potentially impacting students’ motivation and risk of attrition [19, 33]. The current study identified further areas exacerbating the theory-practice gap. Before the clinical placement, students without a healthcare background lacked context for their learning. They lacked understanding of nurses’ shift work and were apprehensive about applying clinical skills learned in the classroom. Hence, some students were uncertain if they were prepared for their first clinical placement or even how to prepare, which increased their anxiety. Prior research has demonstrated that applying theoretical knowledge more seamlessly during clinical placement was supported when students knew what to expect [6]. For instance, a Canadian study exposed students as observers to the healthcare setting before starting clinical placement, enabling early theory to practice connections that minimised misconceptions and false assumptions during clinical placement [34].

In the current study, the theory-practice gap was further exacerbated during clinical placement, where healthcare staff were confused about students’ scope of practice and the course learning objectives and expectations in a postgraduate entry-to-practice nursing qualification. The central booking system for clinical placements classifies first-year nursing students who participated in this study as equivalent to second-year undergraduate nursing students. Such a classification could create a misalignment between clinical educators’ expectations and their delivery of education versus students’ actual learning needs and capacity [3, 31]. Additional communication to healthcare partners is warranted to enhance understanding of the scope of practice and expectations of a first-year postgraduate entry-to-practice nursing student. Educating and empowering students to communicate their learning needs within their scope of practice is also required.

Our research identified a link between students’ personality traits or individual agency and their first clinical placement experience. The importance of a positive orientation towards learning and the nursing profession in preparedness for clinical placement has been highlighted in previous studies [31]. Students’ experience of their first clinical placement in our study appeared to be strongly influenced by their mindset [23]. Some students demonstrated motivation to learn, were happy to ‘roll with the punches’, yet remain active in their learning requirements, whereas others perceived their role as observational and expected supervising nurses to provide learning opportunities. Students who anticipated a passive learning approach prior to their first clinical placement reported boredom, limited activity, and lack of opportunities during their first clinical placement. These students could have a lowered sense of self-efficacy, which may lead to a greater risk of doubt, stress, and reduced commitment to the profession [35]. Self-efficacy theory explores self-perceived confidence and competence around people’s beliefs in their ability to influence events, which is associated with motivation and is key to nursing students progressing in their career path confidently [35, 36]. In the current study, students who actively engaged in their learning process used strategies such as self-reflection and sought support from clinical educators, peers and family. Such active approaches to learning appeared to increase their resilience and motivation to learn as they progressed in their first clinical placement.

Important relationships with supervising nurses, peers, or patients were highlighted in the theme of the reality of navigating placement relationships. This theme links with previous research findings about belongingness. Belongingness is a fundamental human need and impacts students’ behaviour, emotions, cognitive processes, overall well-being, and socialisation into the profession [37, 38]. Nursing students who experience belongingness feel part of a team and are more likely to report positive experiences. Several students in the current study described how feeling part of a team improved self-confidence and empowered work-integrated learning. Nonetheless, compared with previous literature (see for instance, 2), working as a team and belongingness were infrequent themes. Such infrequency could be related to the short duration of the clinical placement. In shorter clinical placements, nursing students learn a range of technical skills but have less time to develop teamwork skills and experience socialisation to the profession [29, 39].

Positive relationships with supervising nurses appeared fundamental to students’ experiences. Previous research has shown that in wards with safe psycho-social climates, where the culture tolerates mistakes, regarding them as learning opportunities, a pedagogical atmosphere prevails [25, 39]. Whereas, if nursing students experience insolent behaviours or incivility, this not only impacts learning it can also affect career progression [26]. Participants who felt safe asking questions were given responsibility, had autonomy to conduct skills within their scope of practice and thrived in their learning. This finding aligns with previous research affirming that a welcoming and supportive clinical placement environment, where staff are caring, approachable and helpful, enables student nurses to flourish [36, 40–42]. Related research highlights that students’ perception of a good clinical placement is linked to participation within the community and instructor behaviour over the quality of the clinical environment and opportunities [27, 28]. Over a decade ago, a large European study found that the single most important element for students’ clinical learning was the supervisory relationship [39]. In our study, students identified how supervising nurses impacted their emotions and this was critical to their experience of clinical placement, rather than how effective they were in their teaching, delivery of feedback, or their knowledge base.

Students’ relationships with patients were similarly important for a successful clinical placement. Before the clinical placement, students expressed anxiety and fears in communicating and interacting with patients, particularly if they were dying or acutely unwell, which is reflective of the literature [2, 10, 11]. However, during clinical placement, relationships with patients positively impacted nursing students’ experiences, especially at the beginning when they felt particularly vulnerable in a new environment. Towards the end of clinical placement, feelings of incompetence, nervousness and uncertainty had subsided. Students were more active in patient care, which increased self-confidence, empowerment, and independence, in turn further improving relationships with patients and creating a positive feedback loop [36, 42, 43].

### Limitations

This study involved participants from one university and a single course, thus limiting the generalisability of the results. Thus, verification of the major themes identified in this research in future studies is needed. Nonetheless, the purpose of this study was to explore in detail the way in which the experiences of clinical placement for student nurses modified initial emotional responses towards undertaking placement and their perceptions of preparedness. Participants in this study undertook their clinical placement in a variety of different hospital wards in different specialties, which contributed to the rigour of the study in identifying similar themes in nursing students’ experiences across diverse placement contexts.

## Conclusions

This study explored the narratives of first-year nursing students undertaking a postgraduate entry-to-practice qualification on their preparedness for clinical placement. Exploring students’ changing perspectives before and during the clinical placement adds to extant knowledge about nursing students’ emotional responses and perceptions of preparedness. Our research highlighted the role that preplacement emotions and expectations may have in shaping nursing students’ clinical placement experiences. Emerging themes from this study highlighted the importance students placed on relationships with peers, patients, and supervising nurses. Significant anxiety and other negative emotions experienced by nursing students prior to the first clinical placement suggests that further research is needed to explore the impact of contextual learning to scaffold students’ transition to the clinical environment. The findings of this research also have significant implications for educational practice. Additional educational support for nursing students prior to entering the clinical environment for the first time might include developing students’ understanding of the clinical environment, such as through increasing students’ understanding of the different roles of nurses in the clinical context through pre-recorded interviews with nurses. Modified approaches to simulated teaching prior to the first clinical placement would also be useful to increase the emphasis on students applying their learning in a team-based, student-led context, rather than emphasising discrete clinical skill competencies. Finally, increasing contact between students and university-based educators throughout the placement would provide further opportunities for students to debrief, to receive support and to manage some of the negative emotions identified in this study. Further supporting the transition to the first clinical placement could be fundamental to reducing the theory-practice gap and allaying anxiety. Such support is crucial during their first clinical placement to reduce attrition and boost the nursing workforce.

## Data Availability

The datasets generated and/or analysed during the current study are not publicly available due to the conditions of our ethics approval but may be available from the corresponding author on reasonable request and subject to permission from the Human Research Ethics Committee.
